# Divergent ocular safety profiles of glucagon-like peptide-1 receptor agonists: a pharmacological heterogeneity framework

**DOI:** 10.3389/fmed.2026.1957367

**Published:** 2026-09-28

**Authors:** Huili Li, Shengke Peng, Daoxi Lei

**Affiliations:** 1 Chongqing College of Traditional Chinese Medicine, Chongqing, China; 2 Department of Ophthalmology, The First Affiliated Hospital of Chongqing College of Traditional Chinese Medicine, Chongqing, China

**Keywords:** diabetic retinopathy, drug-related side effects and adverse reactions, glucagon-like peptide-1 receptor agonists, intracranial pressure, non-arteritic anterior ischemic optic neuropathy, semaglutide, signal transduction

## Abstract

Glucagon-like peptide-1 receptor agonists (GLP-1RAs) have revolutionized the treatment of type 2 diabetes and obesity, yet their ocular safety profile remains contested. The longer-acting semaglutide has been repeatedly linked to an increased risk of non-arteritic anterior ischemic optic neuropathy (NAION) and, in situations of rapid glycemic correction, to early deterioration of diabetic retinopathy, while shorter-acting drugs such as liraglutide and exenatide show much weaker signals. This review explores the pharmacological underpinnings of this difference by combining three lines of evidence: biased agonism and trafficking at the receptor level, cell-type-specific signaling microenvironments, and a recently proposed hemodynamic mechanism based on GLP-1RA-mediated changes in intracranial pressure. The ocular liabilities of GLP-1RAs may be influenced by a multifactorial interplay between ligand-dictated receptor occupancy profiles, cell-type-specific signaling milieu, and individual anatomical vulnerabilities. This model makes testable predictions and has implications for drug choice, risk stratified monitoring, and the development of future agonists with enhanced ocular safety. Key areas for future research include head-to-head comparisons of different GLP-1RAs' signaling in specific ocular cell types and the risk-benefit equation in non-diabetic individuals using these drugs for weight loss.

## 1 Introduction

Glucagon-like peptide-1 receptor agonists (GLP-1RAs) are a mainstay of contemporary metabolic medicine. They not only lower blood glucose, but also lead to significant weight loss, cardiovascular risk reduction and kidney protection (1–5). Semaglutide, a once-weekly agonist with a half-life of ∼165 h, is the most widely used among them due to its greater efficacy in lowering glycated hemoglobin and body weight compared to previous drugs (6, 7). Liraglutide (daily) and exenatide (twice daily or weekly in extended-release form) have shorter durations of receptor occupancy. Dulaglutide (once weekly, half-life ∼90 h) is intermediate in this regard. Global usage is growing rapidly due to new indications, oral preparations and growing use for obesity treatment in non-diabetic patients (8, 9).

There is a clear controversy surrounding ocular safety. In the SUSTAIN-6 cardiovascular outcomes trial, there was a significantly increased incidence of retinopathy complications with semaglutide versus placebo (hazard ratio 1.76, 95% CI 1.11–2.78) (10). In subsequent years, concern expanded to include non-arteritic anterior ischemic optic neuropathy (NAION), an acute, painless, and typically irreversible vision loss resulting from infarction of the anterior optic nerve head (11). Several large-scale observational studies reported an elevated relative-risk signal, with a low absolute incidence, in patients taking semaglutide (12–15). A Danish-Norwegian registry study of more than 61,000 semaglutide initiators reported a pooled adjusted hazard ratio of 2.81 (95% CI 1.67–4.75) compared to SGLT-2 inhibitor initiators, and the risk emerged within the first year of treatment (12). An international pharmacovigilance study covering 180 countries found disproportionately high reporting odds ratios of ischemic optic neuropathy with semaglutide that were much higher than those of dulaglutide or liraglutide (13).

But the evidence is by no means consistent. The LEADER trial (liraglutide) and REWIND trial (dulaglutide) failed to find any significant risk of diabetic retinopathy (16, 17). Someobservational studies have reported no link between exposure to GLP-1RA and NAION (18), and a meta-analysis of randomized controlled trials has found that as a group, GLP-1RAs are not significantly linked to an increase in optic nerve or vision-threatening events (19). Such a discrepancy in safety signals of one agent but not others requires an explanation other than the rapid glycemic correction hypothesis.

The prevailing hypothesis to explain the progression of diabetic retinopathy with GLP-1RA has been the rapid HbA1c reduction or early worsening hypothesis (20, 21). This theory is based on the premise that rapid metabolic improvement disrupts retinal microvascular stability in patients with underlying retinopathy, as observed with intensive insulin treatment and bariatric surgery. Retinopathy signals were confirmed by *post hoc* analyses of SUSTAIN-6 to be concentrated in those with pre-existing diabetic retinopathy, high baseline HbA1c and rapid initial decline in HbA1c (22). This explanation is plausible but does not account for all observations. Why is the NAION signal apparent after nine months of treatment, long after the acute period of glycemic change (12)? Why is the pharmacovigilance signal for NAION so different between semaglutide and other GLP-1RAs, including other long-acting agents (13)? Why does the NAION signal emerge in non-diabetic populations, in whom there are no changes in glycemic control (14)? And why do experimental studies show direct actions of GLP-1RAs on retinal and optic nerve health independent of glycemic control (23, 24)?

An adequate answer requires moving beyond the binary question of whether GLP-1RAs are harmful or protective. The better question is why various GLP-1RAs have divergent ocular effects, and what such divergence tells us about receptor-level pharmacology and tissue-specific signaling. This review combines clinical, pharmacological, and mechanistic data into an integrative model that relates ligand pharmacology, target tissue biology, and host susceptibility, and seeks to produce testable hypotheses and inform clinical decision-making in the absence of definitive prospective data.

## 2 Clinical evidence: a pattern of pharmacological divergence

### 2.1 Diabetic retinopathy: biphasic and context-dependent effects

The interaction between GLP-1RAs and diabetic retinopathy appearsbiphasic and context-dependent. Patients with underlying moderate to severe non-proliferative diabetic retinopathy and poor initial glycemic control may develop transient worsening upon initiation of a potent GLP-1RA like semaglutide in the first 16–26 weeks of treatment (10, 20, 22). The size of the HbA1c reduction seems to be the main factor: a meta-regression of cardiovascular outcome trials discovered that every 0.1 percentage point increase in HbA1c decrease was related to an increased odds ratio of retinopathy events (25), an association at the trial level that does not by itself establish individual-level causality. This effect is not unique to GLP-1RAs, it has been reported with intensive insulin therapy and bariatric surgery, and is mainly due to the susceptibility of a metabolically stressed retinal microvasculature to abrupt changes in glucose availability (20, 21).

Outside this initial 16–26 week time window, the evidence is more neutral or protective. This apparent transition from early worsening to neutral or protective effects is inferred across separate studies rather than demonstrated longitudinally within individual patients, and therefore requires prospective confirmation. The use of GLP-1RA has been linked to a lower risk of progression to proliferative diabetic retinopathy after long-term follow-up (hazard ratio 0.78, 95% CI 0.71–0.86), and lower risks of retinal vein occlusions and neovascular glaucoma in those with existing diabetic retinopathy (26). Using Swedish registry data and Mendelian randomization, Zheng and colleagues reported that treatment with GLP-1RA was associated with a lower risk of diabetic retinopathy (adjusted hazard ratio 0.42, 95% CI 0.29–0.61) and genetically predicted expression of GLP1R was inversely associated with diabetic retinopathy (27). The AngioSafe Type 2 Diabetes study, which combines clinical data from more than 3,000 patients with *in vitro* and *in vivo* angiogenesis assays, did not find an association between GLP-1RA exposure and severe diabetic retinopathy nor a pro-angiogenic effect of exendin-4 (28). These results are consistent with preclinical studies showing that activation of GLP-1R maintains blood-retinal barrier integrity, decreases retinal oxidative stress and inhibits inflammatory cytokine release (29–31).

A meta-analysis by Kapoor and colleagues found that the use of GLP-1RA was linked to a higher risk of early-stage diabetic retinopathy compared to placebo (risk ratio 1.31, 95% CI 1.01–1.68), but a lower risk of late-stage diabetic retinopathy compared to insulin (risk ratio 0.38, 95% CI 0.15–0.98) (32). Albiglutide seemed to be the impetus behind these trends, with agent-specific variations.

The differences between agents are telling. All three GLP-1 receptor agonists (semaglutide, dulaglutide and liraglutide) have shown retinal protection in animal studies, but only semaglutide has produced a consistent early worsening signal in clinical trials (10, 16, 17), although differences in baseline retinopathy, HbA1c reduction, insulin use, study population, and outcome ascertainment limit direct comparisons across cardiovascular outcome trials. This may be due to its greater glucose-lowering efficacy, but it also suggests that the prolonged occupancy of the receptor by semaglutide may lead to different effects on retinal endothelial cells or pericytes that alter the threshold for decompensation during periods of metabolic stress. In a 2026 clinical review, Wu and Chen found ocular risks are clustered in high-risk patients and individualised prescribing with initial ophthalmic evaluation is crucial (33). Similarly, Zhang and Finn, in a 2025 review of all aspects of GLP-1RA ocular effects, concluded that beneficial effects against glaucoma and age-related macular degeneration must be weighed against potential risks for NAION and diabetic retinopathy progression (34). Neither review considered why the risk seems to be specific to semaglutide—a question that demands consideration of pharmacology at the receptor level.

### 2.2 NAION: A signal disproportionately associated with semaglutide

There has been a rapid accumulation of evidence on the association between semaglutide and NAION based on methodologically diverse sources. In an index study using a neuro-ophthalmology referral center database, Hathaway et al. found that the hazard ratio of NAION in patients with type 2 diabetes prescribed semaglutide was 4.28 (95% CI 1.62–11.29) and 7.64 (95% CI 2.21–26.36) in individuals with overweight or obesity, respectively (14). This study was criticized for potential selection bias and because of the high absolute incidence of NAION seen in the semaglutide cohort, which was higher than population-based estimates.

Follow-up population-based studies with national registry data have confirmed the association with smaller effect sizes. A Danish-Norwegian cohort study, which linked national prescription and hospital discharge registries, used an active comparator design, propensity score weighting, and a self-controlled case-series analysis (12). The adjusted hazard ratio of 2.81 (pooled) and absolute risk difference of 1.4 extra cases per 10,000 person-years indicates that the risk is statistically significant but small in absolute terms. Cumulative incidence curves started to separate about nine months after the treatment started- a time trend hard to explain by rapid glycemic correction, as HbA1c decreases are mostly observed within the first 12–16 weeks. The per-protocol analysis produced a significantly larger hazard ratio of 6.35, and of the 32 NAION events originally seen among SGLT-2 inhibitor initiators, 17 were in patients who had later switched to semaglutide–additional evidence that the risk is related to semaglutide exposure and not baseline user characteristics.

The worldwide pharmacovigilance study by Lakhani and colleagues discovered that semaglutide was linked to significantly higher reporting odds ratios of ischemic optic neuropathy compared to metformin, empagliflozin, dulaglutide, and insulin (13). The agreement between two independent spontaneous reporting databases and several comparator drugs is an argument against confounding by indication as the only reason. Kakkar et al. foundthat semaglutide had the strongest NAION signal among the incretin-based therapies (reporting odds ratio 94.45), that liraglutide and the dual GIP/GLP-1 receptor agonist tirzepatide showed significant but substantially smaller signals (reporting odds ratio 4.58 and 2.94, respectively), and that no signal was identified for dulaglutide or exenatide (35). In a Danish cohort of more than 424,000 individuals with type 2 diabetes, Grauslund and colleagues reported a hazard ratio of 2.19 with semaglutide and NAION, which is very similar to the Simonsen study (15).

The picture is not uniform. A multinational OHDSI network study reported mixed findings, with a null active-comparator result under a sensitive outcome definition (HR 1.44, 95% CI 0.78–2.68 versus empagliflozin) but a significantly increased risk under a more specific definition (HR 2.27, 95% CI 1.16–4.46), and a self-controlled case-series analysis showing elevated incidence during semaglutide exposure under both definitions (IRR 1.32, 95% CI 1.14–1.54; IRR 1.50, 95% CI 1.26–1.79) (36). A meta-analysis of randomized controlled trials showed that GLP-1RAs as a group were not significantly related to optic nerve or vision-threatening events, but the analysis was underpowered to detect rare outcomes (19). An agreement statement by the North American Neuro-Ophthalmology Society and the American Academy of Ophthalmology recognized the observational evidence but stressed that causality is not established and the absolute risk, should it exist, is extremely low (37). NAION is uncommon and cannot easily be studied except in very large data sets, and diagnostic coding in administrative databases has not been rigorously validated. The consistency of the signal on various study designs, populations, and comparator groups, however, along with the temporal plausibility and intra-class gradient make it harder and harder to reject the association as being completely artifactual.

The explanations of these discrepant results are worth considering. Shorter follow-up, lower statistical power to detect this rare event, or use of diagnostic codes that have not been validated for NAION have generally limited negative studies (19). The meta-analysis by Li and colleagues, although methodologically sound, included trials in which NAION was an adverse event rather than a predefined outcome, and the overall number of events in all trials was low (19). On the other hand, studies that have found a significant association have usually had longer follow-up periods, active control groups that minimize confounding by indication, or the large sample sizes of pharmacovigilance databases that increase the ability to detect rare events (12–15), although active comparators themselves may not be ophthalmically neutral, since the relative risk estimate depends on whether the comparator (for example, an SGLT2 inhibitor such as empagliflozin) has measurable retinal or optic nerve effects — an assumption that has not been formally tested (38). While these differences do not resolve the controversy, they offer an explanation for the apparent contradictions and suggest that prospective studies with prespecified ophthalmic outcomes are needed.

### 2.3 The clinical divergence as a phenomenon requiring explanation

The data point to a consistent pattern that is the key question in GLP-1RA ocular safety: semaglutide produces stronger signals than liraglutide, dulaglutide or exenatide for both NAION and early diabetic retinopathy progression. This intra-class difference—semaglutide showingthe strongest signal, the relative ordering among the other agents differing between studies — is not easily accounted for by differences in glycemic control, as dulaglutide and liraglutide also produce clinically relevant reductions in HbA1c. We explore pharmacological and tissue-level explanations for this below. The data set for dulaglutide's ocular side effects is much smaller than that for semaglutide and liraglutide, and the apparently weaker signal for NAION should be viewed in this context. In Lakhani and colleagues' pharmacovigilance analysis, the reporting odds ratio for ischemic optic neuropathy with dulaglutide was much lower than that for semaglutide in both the FAERS and VigiBase databases (13). Kakkar and colleagues reported that semaglutide showed the greatest disproportionality, with significant signals for liraglutide and tirzepatide but no signal for dulaglutide or exenatide (35). These pharmacovigilance results are the main empirical source of the comparative risk stratification shown in Table 1, but they need to be validated in specific observational studies with confirmed ophthalmic endpoints.

**Table 1:** Comparative pharmacological and clinical profiles of selected GLP-1 receptor agonists.

Feature	Semaglutide	Liraglutide	Exenatide (IR)	Dulaglutide
Dosing frequency	Once weekly	Once daily	Twice daily	Once weekly
Plasma half-life	∼165 h	∼13 h	∼2.4 h	∼90 h
Receptor occupancy pattern(inferred from PK)	Sustained, continuous	Intermittent (daily peaks)	Phasic (multiple daily peaks)	Sustained
Key retinal protective pathways	cAMP-CREB-STING; AnxA2/MMP-9	PI3 K/Akt; anti-inflammatory; pericyte protection	Akt inhibition; ECM remodeling	Limited direct data
NAION signal (pharmacovigilance ROR)†	Strong (ROR 11–94)†	Significant (ROR 4.58)†	No signal†	No signal†
DR worsening signal (RCTs)	Yes (SUSTAIN-6, HR 1.76)	No (LEADER)	No signal identified	No (REWIND)
ICP modulation (preclinical/clinical)	Not directly tested	ICP reduction (animal model)	ICP reduction (animal model; clinical RCT)	Not directly tested
Weight loss efficacy	High (∼15% at 68 weeks)	Moderate (∼6% at 68 weeks)	Moderate	Moderate-high

AnxA2, Annexin A2; CREB, cAMP response element-binding protein; DR, diabetic retinopathy; ECM, extracellular matrix; HR, hazard ratio; ICP, intracranial pressure; IR, immediate release; MMP-9, matrix metalloproteinase-9; NAION, non-arteritic anterior ischemic optic neuropathy; PI3 K, phosphoinositide 3-kinase; RCT, randomized controlled trial; ROR, reporting odds ratio; STING, stimulator of interferon genes. ^†^ROR denotes reporting odds ratio from pharmacovigilance disproportionality analyses (13, 35); these values reflect spontaneous reporting signals rather than comparative clinical risk.

## 3 Pharmacological foundations of differential signaling

### 3.1 Structural and pharmacokinetic heterogeneity

The molecular engineering of semaglutide, liraglutide and exenatide differs in the way they interact with the GLP-1 receptor (Table 1). Liraglutide is protracted by acylation with a C16 fatty acid which facilitates reversible binding to albumin and increases the plasma half-life to ∼13 h (39). Semaglutide contains amino acid substitutions at positions 8 and 34 and lysine 26 acylation with a C18 fatty diacid—modifications that greatly enhance albumin binding and provide almost complete protection against degradation by dipeptidyl peptidase-4, giving it a half-life of ∼165 h (40). The half-life of exenatide in its immediate-release form is only 2.4 h (41). Dulaglutide, a once-weekly Fc-fusion protein, has a half-life of about 90 h (42).

These differences in systemic persistence lead to different receptor engagement patterns. The sustained systemic exposure to once-weekly semaglutide is expected to resultin minimal ligand-free periods at the GLP-1 receptor (although receptor occupancy in the retina, optic nerve head, and choroid plexus has not been directly measured), while the variable plasma concentrations of once-daily liraglutide or twice-daily exenatide result in intermittent receptor activation with ligand-free intervals. The pharmacological effects of continuous vs. intermittent receptor activation go beyond glucose control (43). Although dulaglutide is also a once-weekly formulation, it exhibits a lower NAION signal than semaglutide, indicating that the half-life cannot explain everything. The molecular structure of semaglutide, including its unique acylation and the consequent albumin binding and tissue distribution pattern, may give rise to additional features relevant to its pharmacology in the ocular tissues.

### 3.2 Biased agonism and receptor trafficking

The GLP-1 receptor, like other class B1 G protein-coupled receptors, does not function as a simple on-off switch. Different ligands stabilize different receptor states that preferentially activate intracellular signaling proteins—biased agonism (44, 45). The canonical signaling pathway includes the activation of adenylyl cyclase by G*α*s, cAMP production and the activation of protein kinase A and exchange protein activated by cAMP. GLP-1R activation can also lead to the recruitment of *β*-arrestins, which shut down G protein signaling, internalize the receptor and trigger G protein-independent signaling pathways (43, 45).

The G protein/ *β*-arrestin signaling balance and the kinetics of receptor internalization/recycling differ significantly among various GLP-1RAs (42, 43, 45). Jones et al. showed that agonists that retain the GLP-1R at the plasma membrane by decreasing the recruitment of *β*-arrestin and increasing the rate of dissociation cause more long-term insulin release than agonists that induce rapid internalization of the receptor (43). Novikoff et al. discovered that semaglutide causes unique spatiotemporal trafficking and recycling patterns of GLP-1R as compared to native GLP-1 and some dual agonists (46). Willard et al. demonstrated that tirzepatide has biased signaling at the GLP-1R, with a preference towards cAMP production over *β*-arrestin recruitment, which is associated with less effective receptor internalization (44).

Lymperopoulos and colleagues have taken the thesis that the GLP-1R is a functionally Gs-selective receptor in physiologically relevant conditions, which is not significantly desensitized and sustains long cAMP signals (47). Assuming that sustained cAMP signaling is the default mode of action of GLP-1R *in vivo*, then the trafficking profile of a given agonist, i. e. whether it facilitates or hinders receptor internalization, might be an even more significant determinant of its net biological effect than its potency to generate cAMP alone. Pearce and others showed that receptor activity-modifying protein 3 binds GLP-1R to bias towards calcium mobilization and away to cAMP to increase glucose-stimulated insulin secretion (48). These advances provide a mechanistic basis for understanding the pharmacological heterogeneity that may also account for differential ocular effects.

### 3.3 The AKT signaling landscape: A window into downstream heterogeneity

The phosphoinositide 3-kinase (PI3 K)/AKT/mammalian target of rapamycin (mTOR) pathway is an example of the diversity of signaling through GLP-1RAs. Lin and colleagues showed that semaglutide blocks AKT phosphorylation at both Ser473 and Thr308 in chondrocytes, removing mTOR-mediated inhibition of autophagy and restoring extracellular matrix homeostasis (49). This observation was at odds with reports that liraglutide activates the PI3 K/AKT pathway for cytoprotection, and the authors explicitly addressed the possibility that persistent versus transient receptor activation could explain different effects on AKT signaling.

The downstream signaling in ocular cells is also complex and cell-type specific. Garczorz et al. discovered that the short-acting agonist exenatide decreased AKT phosphorylation at Ser473 in tumor necrosis factor-alpha-stimulated human retinal pigment epithelial (RPE) cells, inhibited downstream mTORC1 effectors such as PRAS40 and S6 ribosomal protein, and suppressed the production of matrix metalloproteinase-9 (50). These effects are similar to those observed with semaglutide-chondrocytes even though a short-acting agonist was used in another cell type. Exenatide had no effect on cAMP levels in RPE cells, indicating that cAMP-independent pathways are dominant in this cell type (50).

He and others, who have studied the long-acting agonists semaglutide and loxenatide in human retinal vascular endothelial cells, discovered that protective effects on mitochondrial integrity and endothelial junction proteins were mediated by a cAMP-CREB pathway that inhibited stimulator of interferon genes (STING) signaling (23). There was no detectable change in AKT phosphorylation in their system.

To further complicate matters, Li and colleagues also showed that the major anthocyanin in blueberry extracts, cyanidin-3-glucoside, rescued ARPE-19 cells from high-glucose-induced cell death by stimulating endogenous GLP-1 release and restoring GLP-1R/Akt signaling (51). In REDD1-deficient ARPE-19 cells treated with high glucose for 72 h, Akt phosphorylation was decreased, and treatment with cyanidin-3-glucoside rescued Akt phosphorylation in a GLP-1R-dependent manner. This is unlike the Akt-suppressive effect of exenatide reported by Garczorz et al. in this cell line (50). One possibility is that the different mode of receptor activation (intermittent, physiological stimulation by endogenous GLP-1 vs continuous pharmacological stimulation) may lead to different downstream signals or feedback regulation. Another possibility is that the difference reflects differences in exposure time and cell context.

AKT is not a single signaling node but a hub whose phosphorylation state is the net result of the activity of multiple upstream regulators, such as PI3 K, mTORC2 and phosphatases. The overall effect of GLP-1R activation on AKT phosphorylation in any particular cell type depends on the relative expression and activity of these regulators, co-stimulatory signals, and the mode, duration and amplitude of the upstream signal—itself determined by the receptor occupancy and trafficking profile of the agonist, and, as suggested by the comparison between Garczorz and Li, whether the receptor is occupied by endogenous or exogenous agonist. Such diversity of signaling highlights the need for systematic side-by-side comparisons of various GLP-1RAs in specific ocular cell types under identical conditions.

## 4 Tissue-Specific signaling in ocular structures

### 4.1 Retinal endothelial cells and pericytes: barrier protection through divergent mechanisms

The expression of GLP-1R in retinal vasculature has been a controversial issue because of the inconsistent antibody specificity (52). Single-cell RNA sequencing has given a more accurate image. He and others have found GLP-1R mRNA in retinal vascular endothelial cells, dendritic cells and T cells, and validated protein expression with shRNA-mediated knockdown controls (23). GLP-1R was colocalized to a large extent with isolectin B4, an endothelial marker, in fibrovascular membranes of patients with proliferative diabetic retinopathy. In diabetic conditions, retinal endothelial cells show downregulation of GLP-1R—a pathological feedforward process that is also seen in chondrocytes under inflammatory stress (23, 49).

The downstream effects of GLP-1R activation in retinal endothelial cells are the maintenance of mitochondrial integrity, inhibition of cytosolic mitochondrial DNA release and inhibition of the cGAS-STING pathway (23). Treatment with GLP-1RA in diabetic mice decreased TBK1 and IRF3 phosphorylation, maintained endothelial junction proteins, and enhanced vascular morphology and electroretinographic parameters. Semaglutide selectively induces basement membrane remodeling by an Annexin A2-dependent process, restoring the expression of Annexin A2 in retinal endothelial cells, increasing matrix metalloproteinase-9 activity, and stimulating degradation of pathologically thickened collagen IV (53).

In addition to endothelial cells, retinal pericytes are another important target. Lin et al. showed the expression of GLP-1R in primary bovine retinal pericytes and that liraglutide, via activation of GLP-1R, significantly reduced advanced glycation end products (AGEs)-induced pericyte migration through the PI3 K/Akt pathway, reversing the AGEs-induced changes in phosphorylated Akt, phosphorylated focal adhesion kinase, vinculin and matrix metalloproteinase-2 levels (54). This is different from semaglutide's Annexin A2/MMP-9-dependent effects on the endothelial basement membrane, and is an example of how different GLP-1RAs activate different protective mechanisms in the same vascular unit.

These results confirm that GLP-1RAs have direct, glucose-independent protective effects on the retinal microvasculature by different mechanisms depending on the agent and cell type. But they don't explain why semaglutide produces the greatest signals for both NAION and early diabetic retinopathy progression. The protective (and possibly harmful) effects may work through different mechanisms over different time scales—protection of the endothelial barrier over weeks to months, while the hemodynamic effects described in Section 4.3 open the door during the first few weeks of treatment. Or, the overall effect in an individual patient may depend on the balance between protective signals from retinal vascular cells and pro-ischemic effects at the optic nerve head.

### 4.2 Retinal pigment epithelium: ECM remodeling and divergent pathway engagement

The RPE constitutes the outer blood-retinal barrier and is critical to the survival of photoreceptors, visual cycle and immune privilege. ARPE-19 cells express functional GLP-1R, and stimulation of GLP-1 activates protein kinase B and extracellular signal-regulated kinases 1/2 via a PI3K- and epidermal growth factor receptor transactivation-dependent pathway (55).

The RPE is of special interest to the pharmacology of GLP-1RA due to its dual role in maintaining barriers and extracellular matrix (ECM) remodeling. Garczorz et al. demonstrated that exenatide regulates the balance between matrix metalloproteinases and their tissue inhibitors in tumor necrosis factor-alpha-stimulated RPE cells, decreasing MMP-1 and MMP-9 and increasing MMP-2 and partially reversing the inhibition of TIMP-2 (50). This profile indicates that the activation of GLP-1R in the RPE promotes a decrease in net proteolytic activity which may slow down the degradation of Bruch's membrane. Tsou et al. showed that liraglutide alleviates free fatty acid-induced dysfunction of RPE by activating AMPK and restoring lipid homeostasis (56).

RPE cells have a different signaling architecture than retinal endothelial cells. Garczorz et al. found that there was no increase in cAMP levels in RPE cells following activation of the GLP-1R (50), while protective effects observed in retinal endothelial cells by He et al. were dependent on a cAMP-CREB pathway (23). This cell-type specificity supports the case that the net ocular effect of a particular GLP-1RA cannot be extrapolated based on its pharmacology in one cell type.

### 4.3 The optic nerve head and intracranial pressure dynamics

The optic nerve head is a unique anatomical site at the junction of intraocular and intracranial spaces. The prelaminar and laminar portions are exposed to intraocular pressure (IOP) anteriorly, whereas the retrolaminar portion is bathed in cerebrospinal fluid (CSF) within the optic nerve sheath and exposed to intracranial pressure (ICP) posteriorly (11). The central retinal artery and vein run through the subarachnoid space in the intraorbital part of the optic nerve, bringing them into close contact with the CSF-filled sheath.

Lee and colleagues hypothesized a mechanism by which GLP-1RA therapy leads to impaired optic nerve head blood flow (57). They found a tendency for increased retinal arteriovenous transit time during treatment with GLP-1RA, which resolved after stopping treatment in a small cohort of patients. In mice with wild-type genes treated with liraglutide, they observed delayed retinal arterial filling, prolonged arteriovenous transit time, decreased scotopic b-wave amplitudes and increased hypoxia-inducible factor-1 alpha expression. These were associated with a marked decrease in ICP at day 7 of treatment and a decrease in the diameter of the retinal veins.

There is an increasing amount of evidence that GLP-1RAs modulate ICP. The choroid plexus epithelium expresses GLP-1 receptors, which when activated regulate the secretion of CSF by acting on ion transporters such as NKCC1 (58, 59). Botfield et al. have shown that exendin-4 decreased the activity of Na+/K + -ATPase in choroid plexus cell cultures and decreased ICP in a rat model of hydrocephalus in a GLP-1R-dependent way (58). In a randomized clinical trial of idiopathic intracranial hypertension (IIH) with telemetric monitoring, Mitchell and colleagues validated significant ICP decrease by exenatide (59). Clinical benefits in IIH, such as significantly reduced risks of papilledema (risk ratios of 0.25–0.40), visual disturbances, and headache, have been repeatedly confirmed by systematic reviews and meta-analyses, and seem to be independent of weight loss (60–62). In a propensity-matched cohort study, Debiec and colleagues discovered that the use of GLP-1RA was linked to lower odds of needing venous sinus stenting or ventriculoperitoneal shunting (63). In a 2025 review, Chen and Mollan observed that a GLP-1 receptor agonist decreased ICP without weight loss in a phase II randomized controlled trial (64).

The pharmacology of the GLP-1R-mediated ICP regulation is not completely resolved. Jensen and colleagues found that intracerebroventricular (central) injection of exendin-4 in rats stimulated CSF secretion and raised ICP, but peripheral injection did not (65). These results indicate that GLP-1R can be expressed on both luminal (CSF-facing) and basolateral (blood-facing) surfaces of the choroid plexus, with activation of the receptor on each side having opposite effects—a finding that is consistent with the polarized expression of other G protein-coupled receptors in transporting epithelia. Luminal stimulation can lead to CSF secretion, whereas basolateral stimulation, which is the route accessed by peripherally administered GLP-1RAs in clinical practice, may inhibit it. The difference between acute and chronic exposure could also be a factor in the divergent findings. A systematic review by de Oliveira and colleagues highlighted that data on ICP are still variable and that the level of evidence certainty is low (66).

The suggested pathway of ICP modulation to NAION is as follows. The decrease in CSF production caused by GLP-1RA decreases ICP, which decreases the size of the optic nerve subarachnoid space. The resulting sheath narrowing may compromise perfusion of the anterior optic nerve head, which is supplied primarily by the short posterior ciliary arteries [the circulation principally implicated in NAION (11, 67)], and may also affect the central retinal artery within the intraorbital optic nerve (57). Such hemodynamic compromise, which would be subclinical in the majority of people, may be important in those with a crowded optic disc—the structural risk factor that has been established to occur in NAION. Reversibility of retinal perfusion changes after treatment discontinuation has been observed in a small cohort (57); this observation cannot be extrapolated to NAION itself, which commonly produces persistent visual loss, and the related claim that NAION risk decreases after stopping treatment requires direct supporting evidence.

There are a number of strengths of this hemodynamic hypothesis. It describes the intra-class gradient in NAION risk: long-acting agonists would cause more sustained hemodynamic stress on the optic nerve head than the variable effects of shorter-acting agents. It explains the timescale of the emergence of NAION—on the order of months, as would be expected with slow decrease in CSF volume. It allows the fact that NAION may develop in the absence of systemic hypotension, because the mechanism is an anatomically specific effect. This idea is further supported by the observation made by Böhm and colleagues that liraglutide maintains endothelial function in ophthalmic arteries regardless of systemic hemodynamics (68).

The hypothesis has not been proven. Lee and colleagues had a clinical cohort of only nine patients, and the animal experiments were conducted using liraglutide and not semaglutide (57). The correlation between ICP reduction and retinal perfusion can be nonlinear, i. e., there is a threshold below which autoregulatory mechanisms are ineffective. It is not known whether the magnitude or kinetics of the ICP-lowering effect varies between long-acting and short-acting GLP-1RAs, but sustained receptor occupancy of semaglutide would presumably lead to more sustained ICP lowering. The relationship between semaglutide and NAION is also justified by the fact that the observational evidence is consistent regardless of the correctness of the ICP hypothesis: the ICP mechanism is currently the most mechanistically plausible yet unproven explanation of the intra-class gradient.

Another point to consider: GLP-1RAs have systemic vascular effects—blood pressure lowering, endothelial function improvement, anti-inflammatory—that would be expected to decrease, rather than increase, the risk of ischemia. The reason for this lack of effect on NAION in susceptible patients is unclear. The ICP-mediated hemodynamic effect is restricted to the optic nerve head and may not be offset by systemic benefits that are more likely to affect other vascular beds. Systemic vascular improvements take months to years to manifest, while ICP reduction occurs rapidly (days to weeks) —potentially leaving a time gap during which local hemodynamic stress could predominate. Patients with anatomical crowding may have impaired autoregulation and thus even small decreases in perfusion pressure might overwhelm it. These factors do not disprove the ICP hypothesis but emphasize the complexity of NAION risk in patients taking GLP-1RAs.

## 5 Toward an integrative framework

### 5.1 A three-axis model of GLP-1RA ocular effects

The data discussed above suggests a model whereby the ocular effects of GLP-1RAs are dictated by the interaction of three axes of variability. The first axis is ligand pharmacology: the structural characteristics of each GLP-1RA that dictate its half-life, receptor occupancy pattern, trafficking kinetics and signaling bias. The second axis is target tissue biology: cell-type-specific expression of GLP-1R, architecture of downstream signaling networks and functional role of those networks. Host susceptibility is the third axis: anatomical, metabolic or genetic factors which reduce the threshold of clinically significant tissue injury (Figure 1).

**Figure 1:**
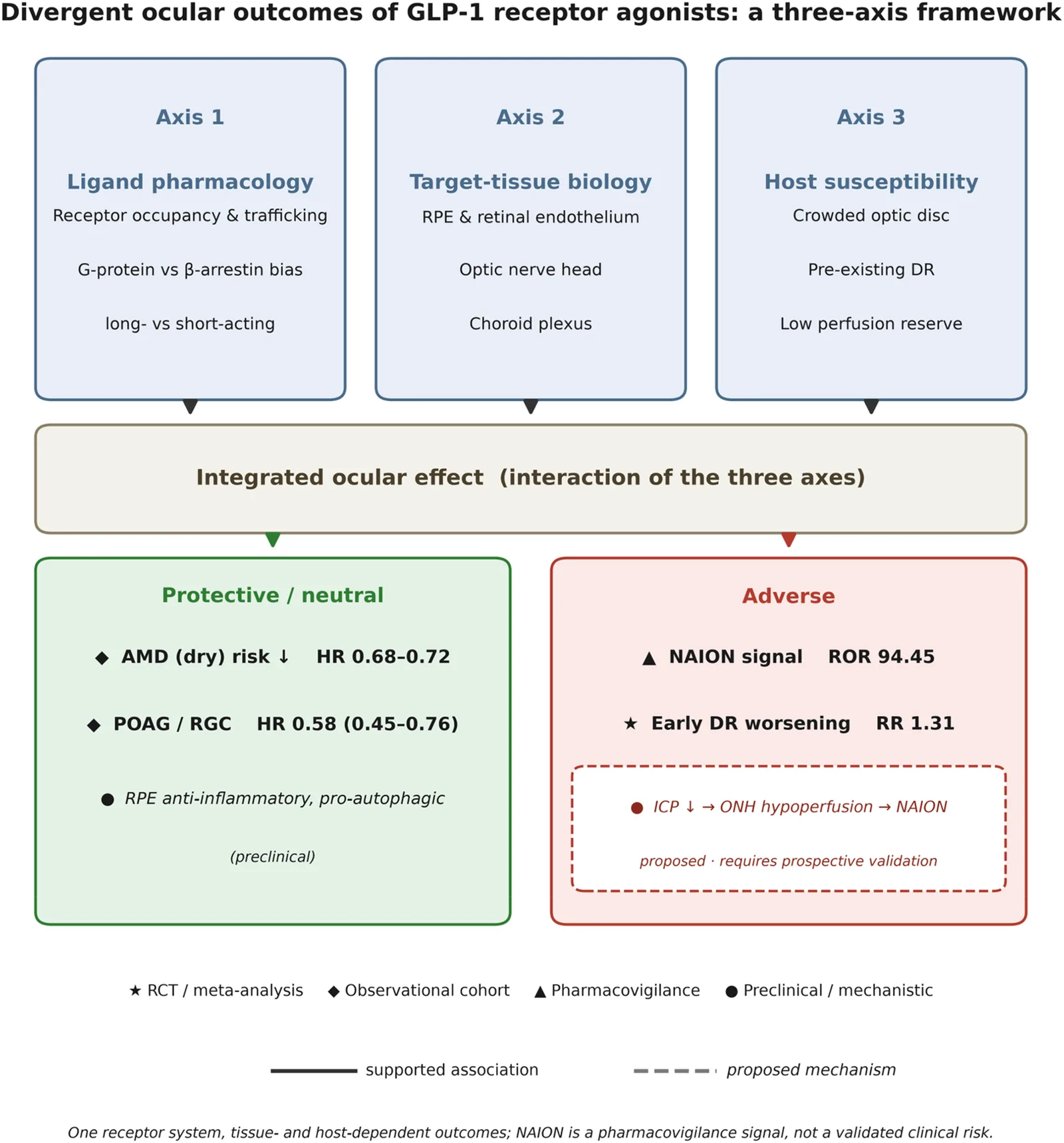
Proposed three-axis framework relating GLP-1 receptor agonist pharmacology to divergent ocular outcomes. The ocular effects of a GLP-1RA are proposed to emerge from the interaction of three axes: ligand pharmacology (receptor occupancy, trafficking, and signaling bias), target-tissue biology (cell-type-specific GLP-1R expression and downstream signaling), and host susceptibility (anatomical and metabolic vulnerability). Solid arrows denote pathways with direct clinical or experimental support; dashed borders denote hypothesized mechanisms, notably the proposed chain from intracranial pressure (ICP) reduction to optic nerve head hypoperfusion and non-arteritic anterior ischemic optic neuropathy (NAION). AMD, age-related macular degeneration; DR, diabetic retinopathy; GLP-1R, glucagon-like peptide-1 receptor; ICP, intracranial pressure; NAION, non-arteritic anterior ischemic optic neuropathy; ONH, optic nerve head; POAG, primary open-angle glaucoma; RGC, retinal ganglion cell; RPE, retinal pigment epithelium.

This model suggests that the unique ocular safety profile of semaglutide may be attributable to its location at the far end of the first axis, continuous receptor occupancy leading to sustained signaling and sustained ICP reduction, acting on tissues of the second axis, retinal microvasculature and optic nerve head, which in susceptible individuals of the third axis have a limited ability to adapt to this stress.

Another example of the tissue-specificity of GLP-1RA pharmacology is the difference between the risk of NAION and neuroprotective effects in glaucoma models. Allan et al. discovered that GLP-1RAs had a significantly lower hazard of primary open-angle glaucoma than insulin (HR 0.58, 95% CI 0.45–0.76) (69). Mouhammad et al. showed that semaglutide rescued retinal ganglion cells against rotenone-induced degeneration by promoting glucose metabolism (70). Sterling et al. demonstrated that the GLP-1R agonist NLY01 decreased retinal ganglion cell death in an ocular hypertension model due to preventing the conversion of astrocytes to a neurotoxic A1 phenotype (71). These neuroprotective actions mediated by the cAMP-PKA-CREB axis and anti-inflammatory pathways are a mechanistically different dimension. Whereas NAION can be caused by hemodynamic compromise, protection of retinal ganglion cells acts directly on neuronal and glial metabolism. The hemodynamic effect may dominate in people with optic nerve head crowding; the neuroprotective effect may dominate in those with neurodegenerative susceptibility.

The model makes testable predictions. Semaglutide's risk of ocular side effects may be shared by other GLP-1RAs with similar receptor occupancy profiles. Patients with crowded optic nerve heads, severe diabetic retinopathy or low retinal perfusion reserve will be most vulnerable. The risk of NAION will be greatest in the first year of treatment, when ICP lowering is greatest, and will diminish over time—as suggested by the Danish-Norwegian survival curves (12). Whether the risk of NAION can be modified by slower dose escalation, the avoidance of concurrent antihypertensive treatment, or pharmacological offsetting of ICP reduction remains an open question that requires prospective investigation; we present it here as a research question rather than as a clinical recommendation.

### 5.2 The Non-diabetic population: an uncertain risk–benefit calculus

The safety of GLP-1RAs for the eyes in non-diabetic patients using these drugs for weight loss is poorly understood. This is a large and rapidly expanding population, but there are few ocular safety data. In the Danish-Norwegian study, the *post-hoc* analysis of semaglutide users without diabetes identified eight NAION events among 156,937 users, which translates to an incidence rate of 0.53 per 10,000 person-years (12). The results were inconclusive because of short follow-up, but the rate was not zero—a critical gap given the widespread use.

The risk-benefit calculation is different to that of type 2 diabetes. Semaglutide has a 26 percent relative risk reduction in major adverse cardiovascular events in patients with diabetes and established cardiovascular disease, which translates to an absolute risk reduction of about 2.3 percent after two years (10). The risk increase of NAION is about 0.01–0.03 percent per year on an absolute basis. In non-diabetic population, the data on cardiovascular outcomes are less developed, and the main advantages are weight loss and metabolic amelioration. The risk-benefit ratio can thus be less favourable but still acceptable to most patients due to the extremely low absolute risk. This uncertainty highlights the necessity of specific ocular safety trials with systematic ophthalmic endpoints and extended follow-up.

### 5.3 Age-Related macular degeneration: potential protective associations

The literature on age-related macular degeneration (AMD) offers a telling contrast to the NAION literature. Several large cohort studies have shown that GLP-1RA use is associated with lower risk of dry AMD. Allan and colleagues reported decreased hazard of nonexudative AMD with GLP-1RAs compared with metformin (HR 0.68), insulin (HR 0.72) and statins (HR 0.70) (69). Ahuja and colleagues, in a study of a non-diabetic obese population, found reduced risk of nonexudative AMD at five years (risk ratio 0.16, 95% CI 0.10–0.28), seven years (risk ratio 0.13) and ten years (risk ratio 0.09) (72). This protective association was confirmed by Myers and colleagues in adults without diabetes (HR 0.47 for nonexudative AMD) (73). Machida and colleagues showed that liraglutide inhibits choroidal neovascularization induced by laser injury through suppression of NLRP3 inflammasome signaling (74). However, a large multinational OHDSI network study found no association between semaglutide and incident neovascular AMD (75), providing a useful balance to the predominantly positive reports.

The pathogenesis of AMD includes chronic inflammation, oxidative stress and defective autophagy in the RPE—processes that are alleviated by GLP-1R activation via anti-inflammatory and pro-autophagic mechanisms (76, 77). The optic nerve head, on the other hand, is susceptible to hemodynamic compromise—a mechanism not covered by these protective pathways and which may be aggravated by ICP reduction.

This is not a paradoxical dissociation. It illustrates the tissue-specificity of GLP-1RA drugs. The same agent activates the same receptor in two different ocular tissues, but the result is different because the microenvironment and disease susceptibility are different. In the RPE, activation of GLP-1R has been associated with anti-inflammatory and survival signals that may protect against degeneration in preclinical models. At the optic nerve head, hemodynamic changes associated with ICP modulation may, in vulnerable anatomies, lead to ischemia. Such disparate outcomes reflect the tissue-specific actions of a single receptor system.

## 6 Clinical implications and risk mitigation

A stratified approach according to the three axes of our framework is justified in patients who start GLP-1RA therapy.

In patients with type 2 diabetes who have documented crowded optic discs, a history of NAION in the other eye or moderate to severe non-proliferative diabetic retinopathy and poor baseline glycemic control, a shorter-acting GLP-1RA like liraglutide, or another glucose-lowering drug, may be a better choice than semaglutide. This needs to be balanced against the better efficacy and cardiovascular advantages of semaglutide. Dilated fundoscopic examination with care to optic disc morphology and optical coherence tomography where possible should be part of a baseline ophthalmic evaluation. Patients are to be informed about the symptoms of NAION and advised to seek emergency care in case of sudden loss of vision. In patients who already have diabetic retinopathy, dose titration over time and follow-up by an ophthalmologist within the first six to twelve months is wise. In patients at risk of both NAION and AMD, ocular risk assessment must consider each anatomical compartment separately.

The absolute risk of NAION in patients taking GLP-1RAs to manage their weight without diabetes seems to be very low, and there is no evidence so far that warrants the withholding of these agents. Since the available analyses are inconclusive, patients are advised to be counseled on the symptoms of NAION and clinicians should have a low threshold of ophthalmic referral.

The risk of NAION is low for all patients and should not overshadow the significant systemic advantages of GLP-1RA treatment. The cardiovascular and renal benefits of these drugs result in absolute risk reductions that are one to two orders of magnitude greater than the absolute risk of NAION (10, 78).

## 7 Knowledge gaps and future research directions

### 7.1 Critical evidence gaps

First, there is no direct comparison of signaling effects of long-acting and short-acting GLP-1RAs in the same type of ocular cell under identical conditions. The pharmacological divergence hypothesis would be supported by systematic head-to-head experiments investigating cAMP kinetics, AKT phosphorylation dynamics, and receptor trafficking in retinal endothelial cells, RPE cells, and optic nerve head astrocytes. The comparison between Garczorz and Li shows that it is important to differentiate between endogenous GLP-1 secretion-induced signaling and exogenous agonist-induced signaling.

Second, the ICP hypothesis needs to be prospectively validated in the setting of NAION. Longitudinal ICP measurements, retinal perfusion imaging and patient-reported visual outcomes are required. The conflicting results of Jensen and colleagues highlight the need for further studies of the subcellular localization of GLP-1R in the choroid plexus and the overall impact of systemic administration of GLP-1RAs on CSF dynamics in humans.

Third, the safety of GLP-1RAs in non-diabetic weight-management patients is poorly understood. Specific studies with detailed ophthalmic outcomes and long-term follow-up are needed.

Fourth, the question of whether the ICP-lowering effect varies in magnitude or kinetics across GLP-1RAs has not been directly addressed. Since there is a lot of clinical information on the effects of GLP-1RA in IIH, it is possible to conduct a comparative study with ICP as one of the main outcomes.

Fifth, the hypothesis of optic nerve sheath narrowing and central retinal artery compression due to ICP reduction needs to be directly tested by imaging studies that combine optic nerve sheath diameter measurement with optical coherence tomography angiography.

### 7.2 Future directions

Future prospective observational studies with systematic ophthalmic phenotyping should assess the effects of semaglutide, liraglutide and non-GLP-1RA agents in patients stratified by optic disc morphology and diabetic retinopathy. Pharmacoepidemiological analyses should determine if NAION risk is modified by factors affecting ICP or CSF flow. The ocular safety of dulaglutide also merits further study, as there is less information available on this agent.

Long-term, the concept of biased agonism could be used to develop safer GLP-1RAs for the eye. If the effect on ICP is mediated by a specific signaling pathway, it may be possible to design compounds that are metabolically active but have reduced effects on CSF and retinal blood flow. The discovery of receptor activity-modifying protein 3 as a modulator of GLP-1R signaling bias (48) provides one strategy to achieve tissue-specific drug actions.

## 8 Conclusion

GLP-1 receptor agonists are one of the most significant therapeutic breakthroughs in metabolic medicine over the last 20 years. Their increasing indications make it crucial that their safety profile be known accurately. The correlation of semaglutide with NAION, and the risk of early progression of diabetic retinopathy are valid safety issues. But the tendency to frame these concerns as a class-wide phenomenon hides the pharmacological and biological complexity at play.

Differences in ocular safety signals may reflect genuine differences in the interaction of GLP-1RAs with their receptor and the influence of cellular and anatomical factors on the downstream effects of the receptor. Strategies to enhance ocular safety include appropriate drug selection, risk-based monitoring, and agonist design for specific signalling properties. The potential benefits against AMD (proposed to be mediated by anti-inflammatory and pro-autophagic actions in the RPE) are accompanied by risks for NAION (proposed to be mediated by hemodynamic actions at the optic nerve head, although the underlying mechanism remains unproven). These seemingly conflicting outcomes are not mutually exclusive—they may reflect the actions of a single receptor system in different tissues. The path forward requires collaboration across endocrinology, ophthalmology, pharmacology, and epidemiology to transform a clinical controversy into a set of testable hypotheses and, eventually, into evidence-based guidance.
